# The Use of Molecular Profiling to Track Equine Reinfection Rates of Cyathostomin Species Following Anthelmintic Administration

**DOI:** 10.3390/ani11051345

**Published:** 2021-05-09

**Authors:** Alexa C. B. Johnson, Amy S. Biddle

**Affiliations:** Department of Animal and Food Sciences, University of Delaware, Newark, DE 19716, USA; alexaj@udel.edu

**Keywords:** equine, strongyle, anthelmintic, cyathostomin, fecal egg count, resistance, amplicon sequencing

## Abstract

**Simple Summary:**

Cyathostomins (small strongyles) are a multispecies group of intestinal parasites in horses and the main target of deworming efforts by horse owners. It is not known whether species of cyathostomins have individual responses to dewormers. The objective of this study was to identify differences between cyathostomin species in reemergence rates following commercial dewormer treatment. This study used gene sequencing to profile the presence/absence of cyathostomin species in fecal samples at 2-week intervals following deworming to determine how quickly each species reinfected horses. Moxidectin was found to be the most effective at slowing the overall reemergence of these parasites, followed by Ivermectin, then Pyrantel. Seven species were resistant to all three deworming products. This study demonstrates that dewormer sensitivity differs between cyathostomin species, which could lead to more targeted control measures.

**Abstract:**

Cyathostomins are a multispecies parasite ubiquitous in Equids. Cyathostomins have developed resistance to all but one class of anthelmintics, but species-level sensitivity to anthelmintics has not been shown. This study measured reinfection rates of cyathostomin species following the administration of three commercial dewormers. Nine treated horses were compared with 90 untreated controls during June-September 2017–2019. Ivermectin (IVM) (n = 6), Moxidectin (MOX) (n = 8) or Pyrantel (PYR) (n = 8) were orally administered. Fecal samples were collected every 14 d for 98 d. Fecal egg count reductions (FECR) were calculated using a modified McMaster technique. Nineteen cyathostomin species were identified by 5.8S-ITS-2 profiling using amplicon sequencing. Data were analyzed in QIIME1 and R statistical software using presence/absence methods. MOX had the lowest numbers of species present over the time course, followed by PYR then IVM (7.14, 10.17, 11.09, respectively); however, FECR was fastest for PYR. The presence of seven species: *Coronocyclus labiatus, Cyathostomum catinatum, Cyathostomum tetracanthum, Cylicocylus elongatus, Cylicodontophorus bicoronatus, Cylicostephanus minutus,* and *Cylicostephanus goldi* were unaffected by treatment (*p* > 0.05) points to species-specific differences in dewormer sensitivity and environmental persistence. Identifying resistance patterns at the species level will enable mechanistic understandings of cyathostomin anthelmintic resistance and targeted approaches to control them.

## 1. Introduction

Cyathostomins are the most prevalent equine intestinal parasite group comprising 89–100% of the worm burden in horses [1,2]. Over 50 cyathostomin species from 14 genera have been described, and a single horse may harbor from 1 to 26 species at a time [3,4]. Cyathostomins can be found in horses of all ages and as early as 4 months, and while cyathostomin burdens can vary, the presence of the parasitic worms remains constant in the gut for the animal’s entire life [5]. Young horses have higher infective rates due to naïve immune systems when compared to older horses [5] and horses who live on pasture will have higher infective rates from grazing on or near fecal material when compared to stalled horses [6]. Luminal and encysted parasites in the equine gut can cause a myriad of health concerns, including weight loss, poor feed efficiency, dull coat, diarrhea, intermittent colic, and decreased performance [7]. The spontaneous eruption of larvae encysted in cecal or colonic tissue (larval cyathostominosis) carries a 50% fatality rate due to tissue damage [6,8,9,10,11]. In Canada, larval cyathostominosis has been suggested as an emerging equine disease that may presage trends for horses in the United States [8].

Benzimidazoles, tetrahydropyrimidines (pyrantel pamoate (PYR)), and macrocyclic lactones (Ivermectin (IVM) and Moxidectin (MOX)) are the main anthelmintic drug classes used to control cyathostomins in horses [12]. Due to high frequency and prolonged use, the development of resistance to benzimidazole followed by tetrahydropyrimidines has left one effective option, macrocyclic lactones, against all stages of cyathostomins [13,14]; however, resistance to this class appears to be emerging [15,16]. Some cyathostomins even exhibit multidrug class resistance [15,17,18,19].

When MOX, IVM, and PYR were first introduced to the market, the MOX egg reappearance period (ERP) was 16–22 weeks [20,21,22], 9–13 weeks [22,23,24], and 5–6 weeks [24,25], respectively. The ERP of these three anthelmintics is currently reported at 10–12 weeks, 6–8 weeks, and 4–5 weeks, respectively [26].

Four mechanisms have been suggested for the rise of anthelmintic resistance in horses; (1) pre-existing alleles for resistance, (2) spontaneous mutations before or at the time of anthelmintic exposure, (3) frequent mutations for the reappearance of resistant alleles, or (4) host migration of resistant alleles is spread through new populations [16,27,28,29].

Thus far, efforts to understand the mechanisms of resistance of cyathostomins have largely considered them to be a monolithic group [19,30]. It is unlikely that drivers of resistance act uniformly across the 50 cyathostomin species, but little is known about species-specific sensitivity to anthelmintic drugs or the environmental factors favoring the success of individual cyathostomin species [31]. Species and genera contributions to shortened egg reappearance rates have been studied via morphological identification of adult worms [32], PCR-ELISA [33,34], and Reverse Line Blot [35,36,37] techniques. All three of these study techniques are laborious and difficult to conduct on a large number of horses, particularly morphological identification, because horses must be euthanized and necropsied [38]. The AAEP guidelines for the fecal egg count reduction test remain the gold-standard and most widely adopted method to determine anthelmintic resistance [26,39]. However, the interpretation from the FECR to the ERP still varies between researchers and makes comparisons with the literature difficult. While DNA sequencing has been used to identify cyathostomins at the species level since the 1990s [40,41,42], the use of marker genes to survey cyathostomin populations via NGS of fecal material (as is commonly performed for bacteria) is a novel approach.

The objective of this study was to use next-generation sequencing (NGS) to track the presence of cyathostomin species in equine fecal samples following treatment with three commercial anthelmintics: Moxidectin, Ivermectin, and Pyrantel compared with untreated controls. We hypothesize that species-specific differences in response to anthelmintic drugs underly the ability of cyathostomins to develop resistance. This research describes species-level differences in the response and reemergence of cyathostomins to each anthelmintic and demonstrates the efficacy of a noninvasive sequence-based methodology for identifying the presence of cyathostomin species from fecal samples.

## 2. Materials and Methods

### 2.1. Study Design

This experiment was approved by the University of Delaware Animal Care and Use Committee (#AUP90R).

Horses housed at two locations were enrolled in the study (Table 1).

All horses were considered to be idle with occasional pleasure riding, lived in mixed-sex pastures, and had been residents of their respective herds for a minimum of 2 years prior to the study. The horses had not received anthelmintic treatment or antibiotics for 180 days prior to the beginning of the study. All horses were housed in grass pastures with year-round ad libitum access to forage, pasture, water, and mineral salt blocks and received grain supplementation only as needed to maintain body condition. The study tested three different anthelmintics during the summer months (May–September) over 3 years (2017–2019) with Moxidectin (MOX) (n = 8) conducted in 2017, Pyrantel (PYR) (n = 8) conducted in 2018 (n = 8), and Ivermectin (IVM) (n = 6) conducted in 2019. The summer season across all three treatments/years (average 24.15 °C, 11.92 inches precipitation) was fairly equivalent to normal DE summer season conditions of warm and wet conditions (average 24 °C, 12” inches precipitation) [43]. To evaluate the natural fluctuations of cyathostomin species for the duration of the study period, untreated control (CON) fecal samples (n = 90) were collected from pasture-managed horses in the mid-Atlantic region who had not received anthelmintic treatment within the last 180 days parallel to the sampling points of the horses enrolled to the study (Supplementary, Table S1).

### 2.2. Fecal Sample Collection

Pre-treatment control samples were obtained on Day 0, and anthelmintics were orally administered according to the manufacturer’s instructions. Equine weights were estimated using a horse and pony weigh tape (Coburn, Whitewater, WI), and weights were rounded up to prevent underdosing horses. Post-treatment fecal samples were collected every 14 d for 98 d total. Fecal samples were obtained by picking up feces within 5 min of defecation with an inverted Ziploc bag, and the air was expelled. Immediately, approximately 4 mL of fecal material was aliquoted from the inside of a fecal ball using a sterile spoon into a 5 mL tube containing 1 mL of DNA/RNA Shield preservative (Zymo, Tustin, CA, USA) and shaken vigorously. This sample was placed at −20 °C until nucleic acid extraction could be performed (within 1 month). Fecal samples were stored in individual Ziploc bags at 4 °C until the FEC could be performed (within 48 h).

### 2.3. Fecal Egg Count Reduction and Egg Reappearance Period Tests

Fecal egg counts were conducted using the Paracount-EPG Kit (Chalex LLC, Park City, UT, USA) according to the manufacturer’s instructions. Fecal egg count reductions (FECR) [26] were calculated for each individual horse and were determined as:

(1) $$FECR=\left(\frac{FEC_{0d}-FEC_{post\;treatment}}{FEC_{0d}}\right)*100$$

Day 0 FEC (*FEC_0d_*) represents the pre-treatment survey, and FEC conducted on each subsequent sampling day (Day 14, 28, 42, 56, 70, 98) for each treatment was used for *FECpost treatment.* FECR values were averaged for each treatment at each time point, and an FECR cut-off of ≤90% was used for MOX and IVM, and an FECR cut-off of ≤80% was used for PYR as outlined by Nielsen et al. [26] to determine a shortened fecal egg reappearance period (ERP) as a measure of anthelmintic resistance [14,44].

### 2.4. DNA Extraction and Sequencing

DNA was extracted after thawing using a commercial kit (QIAGEN QIAmp Powerfecal DNA Isolation Kit, Germantown, MD, USA). DNA triplicates were tested for quantity and quality using Qubit (Thermo Fisher, Waltham, MA, USA) and Nanodrop (Thermo Fisher, Waltham, MA, USA) according to manufacturer instructions. Amplification of the 5.8S-ITS-2 rRNA and attachment was performed using custom region-specific primers: forward primer 5′-GACTAGCTTCAGCGATGGA-3′ and reverse primer 5′-AACGYTGTCATACAGGCACT-3′. Primers were designed using Primer1 [45] to produce amplicons that could be used on the Illumina MiSeq platform (450–480 basepairs), targeting the highly conserved 5.8S and ITS-2 rRNA gene regions [46,47] of the 19 equine cyathostomins included in this study (Table 2). Primer specificity for equine cyathostomins was validated through morphological and molecular identification of adult cyathostomins from equine feces (unpublished) (Supplementary, Table S2).

PCR products were pooled and sequenced via Illumina MiSeq platform by RTL Genomics (Lubbock, TX). Paired ends were joined using FLASh (v.1.2.11) [48]. Quality filtering was performed in QIIME1 [49] using the split_seqs.py command, and taxonomic assignments were conducted using the map_reads_to_reference.py command with the aligned sequences of 19 cyathostomins (Table 2) using a QIIME 1 [49] pipeline. The identification and naming conventions by Lichtenfiels et al. [50] were used in this study. Sequence data have been submitted to the NCBI Sequence Read Archive within PRJNA716069.

### 2.5. Phylogenetic Analysis

Phylogenetic trees were constructed of the 19 cyathostomin species genomic sequences listed in Table 2. *Strongylus equinus* (KM605251)*, Strongylus vulgaris* (AP017698)*,* and *Syngamus trachea* (GQ888718) were included as outgroups. Bootstrap analysis with 500 replicates was used to assess the confidence limits of the branches of the maximum likelihood trees. Trees were drawn using MEGA7: Molecular Evolutionary Genetics Analysis version 7.0 for bigger datasets: (https://www.megasoftware.net/ accessed on 17 September 2020) [51].

### 2.6. Statistical Analysis and Species Frequency Reductions

Due to variable cell numbers and DNA content for different cyathostomin development stages [52], species abundance estimates could not be made, and data were analyzed using presence/absence methods [38]. All data were evaluated in R statistical software [53]. Cyathostomin species frequency of presence was determined by the percentage of horses harboring the species at each given time point and treatment and analyzed with ANOVA and Tukey all-pair comparison method with significance determined at (*p* ≤ 0.05) and a tendency toward significance at (0.05 ≤ *p* ≥ 0.10). Cyathostomin species frequency of presence reductions (SFPR) was determined as:

(2) $$SFPR=\left(\frac{SFP_{0d}-SFP_{post\;treatment}}{SFP_{0d}}\right)*100$$

Cyathostomin species frequency of presence (*SFP_0d_*) represents the pre-treatment survey, and SFP of each subsequent sampling day (Day 14, 28, 42, 56, 70, 98) for each treatment were used for *SFPpost treatment.*

To predict the probability of a species’ presence at each given time point post-treatment (MOX, IVM, and PYR), binomial logistic regression models were employed compared to the CON samples. Spearman’s coefficient, r, was used to determine correlations between cyathostomin species and anthelmintic treatment with significance determined at (−0.3 ≤ r ≥ 0.3). The Spearman correlation, r, is considered to be fairly significant at (≥+/−0.3–<+/−0.5), moderate with (≥+/−0.5–<+/−0.7), strong with (≥+/−0.7–<+/−0.9), and substantial with (≥+/−0.9–+/−1.0).

## 3. Results

### 3.1. Sequencing Results

Amplicon sequencing yielded an average of 14,773 joined reads per sample (standard deviation = 5699). One horse was removed from the MOX and PYR trials due to inadequate amplification for more than 50% of the time points. *POT. imparidentatum* was not observed in this study and is among the less common cyathostomin species and was removed from the study [5]. *CS. tetracanthum* was observed at a very low rate and is also a lesser found species but was not removed from the analysis. *CS. teteracanthum* was observed at Day 0 and 14 of the CON samples but was then no longer detected in any samples except for Day 70 during MOX treatment in 38% of the samples (Supplementary, Table S3).

### 3.2. Fecal Egg and Species Count Reductions

FECR tests revealed an ERP of 100% 14 d post-administration for both MOX and IVM and 98.37% for PYR. PYR reached a shortened egg reappearance rate (ERP < 80%) by 28 d, followed by IVM at 42 d and MOX at 84 d (ERP < 90%) (Table 3). The overall total number of species observed in the fecal material was reduced the most by MOX, followed by IVM and PYR (Table 3). On average, horses enrolled in the study harbored 9.39 (±3.16 s.d.) species at 0 d (Supplementary, Table S4).

**Figure 1:**
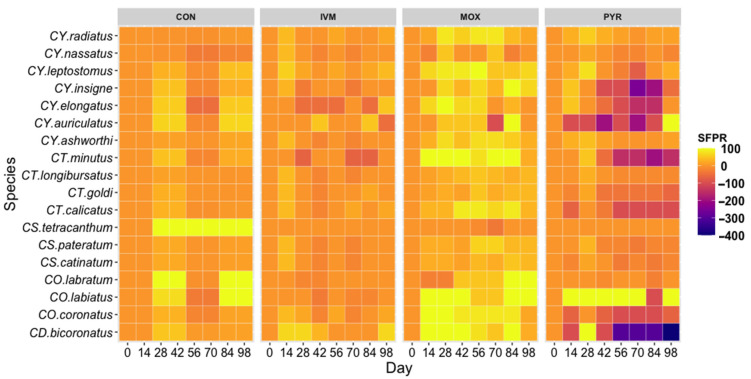
Heatmap of cyathostomin SPFR to anthelmintic treatments. SFPR demonstrates the natural variation of species populations in the CON group and how the species, in turn, respond to anthelmintic treatment. CON-control, IVM-Ivermectin, MOX-Moxidectin, PYR-Pyrantel.

Natural fluctuations of cyathostomin species presence was demonstrated by the CON group, which showed that 12 species were reduced at Day 28–42, then rose back to 0 d infection rates at 56–70 d but then reduced again at 84–98 d (Figure 1). Only five species *(CY. radiatus, CY. nassatus, CY. ashworthi, CT. longibursatus, CS. catinatum)* in the CON samples appeared to be consistently present and not demonstrating this natural environmental response.

Six species demonstrated IVM resistance (*CY. elongatus, CY. auriculatus, CT. minutus, CS. tetracanthum, CO. labratum, CO. labiatus*), showing no reduction at 14 d post-treatment (Figure 1). IVM reduced species infection rates for 11 species at 14 d post-treatment, but a 100% reduction in any species was not observed, as was achieved by MOX and PYR (Figure 1). Cyathostomin species treated with IVM returned to 0 d infection rates or greater by 28 d in all species except for *CY. leptostomus* (42 d), *CO.coronatus* (42 d), and *CD. bicoronatus* (56 d). The extended period of *CD. bicoronatus* reduction may be a factor of environmental and natural species patterns of the region as reflected in the CON group. MOX was able to achieve 100% reduced SFP rates in four species 14 d post-treatment (*CT. minutus, CO. labiatus, CO. coronatus, CD. bicoronatus*) and continue to reduce rates for at least four weeks. Five species (*CY. leptostomus, CY. insigne, CY. elongatus, CY. auriculatus,* and *CS. catinatum)* were reduced by 100% at later time points but were able to reinfect horses more quickly than the four species showing more sensitivity toward MOX (Figure 1).

### 3.3. Anthelmintic Resistance

Over the entire course of the study, seven species, *CO. labiatus* (*p* = 0.403)*, CS. catinatum* (*p* = 0.066)*, CS. tetracanthum* (*p* = 0.281), *CY. elongatus* (*p* = 0.106)*, CD. bicoronatus* (*p* = 0.108)*, CT. minutus* (*p* = 0.074) and *CT. goldi* (*p* = 0.189), exhibited multidrug resistance to all three anthelmintics (Treatment, *p* > 0.05) (Table 4).

**Figure 2:**
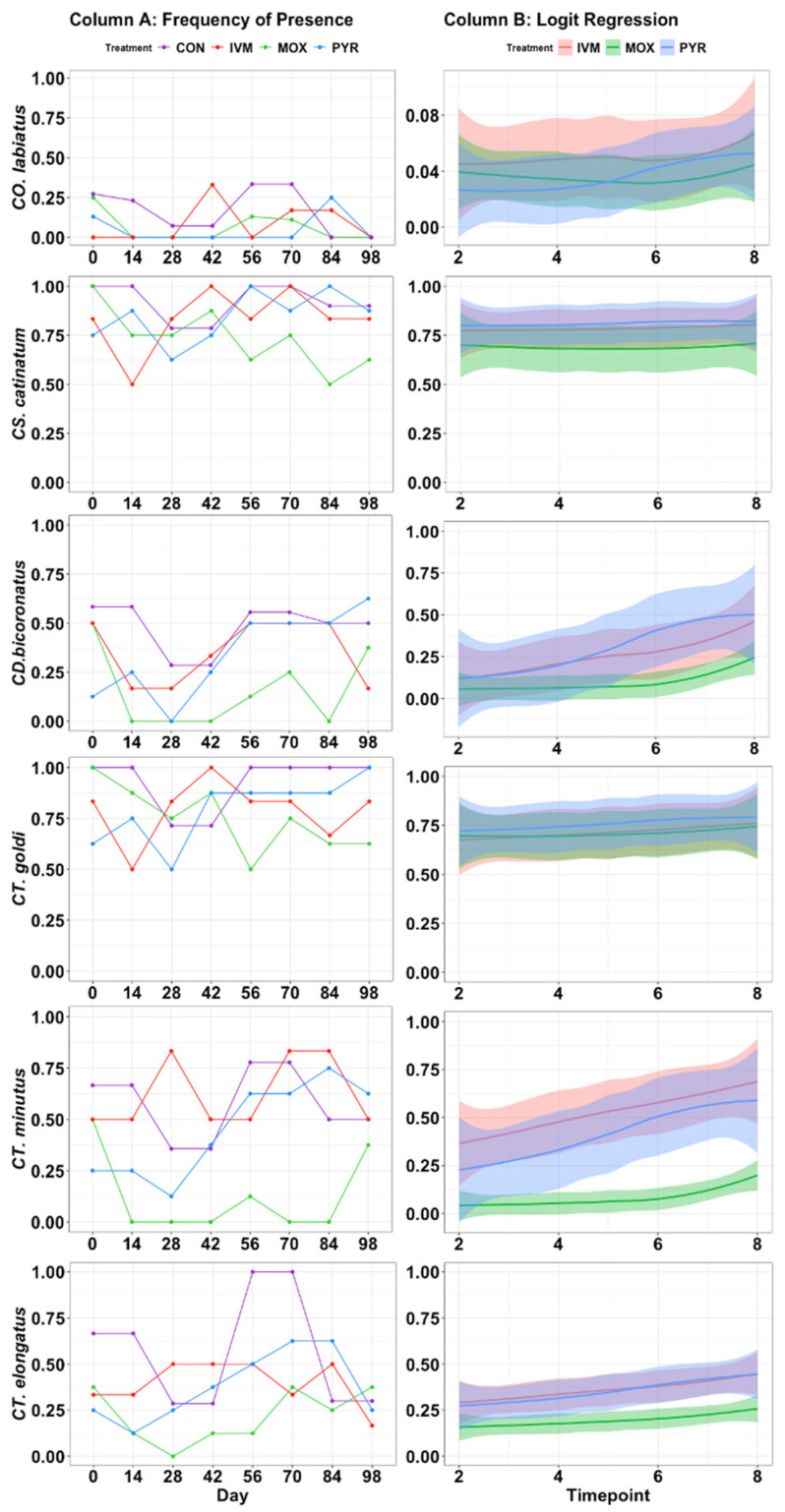
Frequency of presence and logit regression models of the multidrug-resistant species presence in the study herd. Column A contains frequency of presence at each time point, and column B contains the binomial logistic regression predicted probability of presence plots based on CON samples. Timepoint 2 = Day 14, Timepoint 3 = Day 28, Timepoint 4 = Day 42, Timepoint 5 = Day 56, Timepoint 6 = Day 70, Timepoint 7 = Day 84, Timepoint 8 = Day 98. Shaded areas indicate 95% confidence intervals.

These seven species appear to be acutely responsive to treatment, but the quick reinfection is a demonstration of multidrug resistance (Figure 2, Column A). Frequency of presence infection rates were used to predict the species’ ability to reinfect the herd (Figure 2, Column B).

*CS. catinatum* and *CT. goldi* demonstrated the highest level of anthelmintic resistance with a ≥50% chance of observing these species in the herd 14 d post-treatment and no change in the probability of infection regardless of treatment or time (Figure 2, Column B). Three species, *CO. labratum, CY. auriculatus,* and *CT. longibursatus* showed a tendency to develop multidrug resistance (*p* (0.5 ≥ *p* ≤ 0.10)), and multidrug resistance could not be detected in the last seven species (*p* ≥ 0.10) (Table 4).

Tukey’s all-pair comparison testing determined that MOX reduced 10 species populations (CO. coronatus, CY. ashworthi, CY. insigne, CY. leptostomus, CY. nassatus, CY. radiatus, CY. elongatus, CD. bicoronatus, CT. calicatus, CT. longibursatus, and CT. minutus) and PYR reduced four species (CO. coronatus, CY. insigne, CY. leptostomus, and CT. calicatus) when compared to the CON group (*p* < 0.05) but none could be determined to be reduced following IVM treatment (Table 4).

Spearman correlation testing revealed that eight species (CO. coronatus, CY. ashworthi, CY. insigne, CY. leptostomus, CY. nassatus, CY. radiatus, CT. calicatus, and CT. minutus) were negatively correlated with MOX treatment (Table 4), while CO. labratum was found to be positively correlated.

### 3.4. Phylogeny

**Figure 3:**
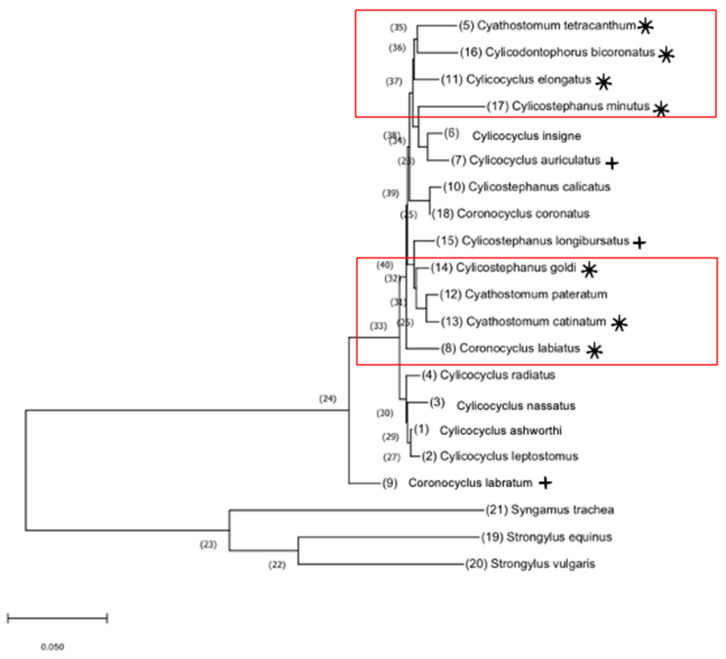
Phylogenetic relationships of equine cyathostomins based on full-length gene sequences. Maximum likelihood tree using bootstrap inference of equine cyathostomins in this study (accession numbers listed in Table 2). *Syngamus trachea* (KM605251)*, Strongylus equinus* (AP017698)*,* and *Strongylus vulgaris* (GQ888718) were included as outgroups. * indicates resistance to IVM, MOX, and PYR (*p* ≥ 0.05). + indicates trending resistance (0.01 ≥ *p* < 0.05). to IVM, MOX, and PYR. Red boxes indicate the clustering of species presenting resistance.

The maximum likelihood tree showed that the seven species demonstrating multidrug class resistance (*CO. labiatus, CS. catinatum, CS. tetracanthum, CY. elongatus, CD. bicoronatus, CT. minutus,* and *CT. goldi)* form two closely related clades (Figure 3). The three species that showed a tendency toward multidrug resistance did not group closely together, although *CY. auriculatus* and *CT. longibursatus* emerged between the two highly resistant clades.

## 4. Discussion

Using noninvasive sequence-based methodology for identifying the presence of cyathostomin species from fecal samples, this research demonstrates species-specific differences in the response and reemergence of cyathostomins to three commonly used anthelmintics. This work shows the efficacy of NGS strategies for profiling cyathostomins from fecal samples and challenges the prevailing strategy of treating these parasites as a monolithic group.

### 4.1. Fecal Egg Count Reductions and Species Frequency of Presence Reductions

While cyathostomin species variation is observed between study populations due to geography and climate, a globally recognized ‘core’ group of 10–12 species (*CS. catinatum, CS. pateratum, CO. coronatus, CO. labiatus, CO. labratum, CY. nassatus, CY. leptostomus, CY. insigne, CT. longibursatus, CT. goldi, CT. calicatus, CT. minutus)* has been recognized that comprises up to 99% of the cyathostomin burden in horses [4,13,54,55,56,57,58,59]. While the methods used in this study cannot measure the species composition of the parasite burden of individual horses, we did observe that five species of the ‘core’ group (*CT. longibursatus, CS. catinatum, CS. pateratum, CY. ashworthi, CT. goldi*) plus an additional two species (*CY. radiatus,* and *CY. nassatus)* were found in ≥80% of all CON samples (Supplementary, Table S5). The high prevalence rates of *CY. radiatus,* and *CY. nassatus* may be a result of the small sample size used in this study.

Cyathostomin transmission is known to be seasonally regulated [55,60] based on both larvae and adult worm preferences for temperature and moisture. The optimum temperature for the development of strongyle eggs and larvae ranges from 25–33 °C [1,3,55] with an upper limit of 38 °C [61] and the optimal fecal moisture level of the closely related ruminant trichostrongylids is 57–63% with larval development not occurring below 20% [62,63]. The cyathostomin lifecycle poorly tolerates desiccation [55] and freeze/thaw cycles [64,65]. This study demonstrated through the CON samples that a natural species-specific temporal response specific to the mid-Atlantic region could be observed. The fluctuation of infectivity rates between cyathostomin species could indicate increased or decreased abilities to adapt to environmental conditions to ensure survival in addition to their heightened anthelmintic resistance responses. This study did not experience any temperature or wet conditions outside of the optimal range reported for cyathostomins.

This study observed similar ERP for MOX and IVM to the ERP reported elsewhere [26]; however, our results observed a shorter ERP for PYR than previous reports [21]. The shortened ERP for PYR could be a reflection of a heightened herd specific response; however, the horses enrolled in the study did not have an extensive history of PYR treatment, and the FECR for PYR at 14 d is higher than reported in other studies that reported values of 87.1% [66] and 73.4% [15], which makes this explanation unlikely. These results may indicate that the ERP for PYR has been further reduced since reported by the AAEP Parasite Control Guidelines [26].

Anthelmintic resistance has been perpetuated by selective pressure caused by overuse and overexposure to anthelmintics. Therefore, it is expected that the species that are known to be the first infectors and members of the ‘core’ harbor the highest levels of resistance traits. In Kentucky, USA [35], foals and yearlings were found to already be infected with IVM-resistant cyathostomins (*CY. nassatus, CT. longibursatus, CT. calicatus,* and *CT. minutus),* and another foal study [67] found that the highly resistant species, *CS. catinatum* and *CT. goldi,* are among the first to infect foals suggesting similar patterns of species-specific resistance observed in this study.

This study also demonstrates differences between drug efficacy and parasite resistance as defined by Barnes et al. [55] and Dargatz et al. [68,69]. In general, anthelmintics with poor absorption rates (such as tetrahydropyrimidines (PYR)) present cyathostomins with less selective pressure to develop resistance than drugs with higher absorption rates (macrocyclic lactones (IVM and MOX)) [70]. Using targeted deworming strategies such as using drugs with lower absorption rates and treating horses with the highest FEC serves to preserve a refugia of less resistant parasites for the population as a whole [26,70]. The present study observed that PYR had a low efficacy overall (indicated by ERP and total species prevalence rates) and low resistance since 100% reduced SFP rates in some species were observed. On the other hand, IVM had higher efficacy and a higher level of resistance observed by low SFP rates. IVM demonstrated a 100% FECR as expected; however, it was unexpected to observe no differences between CON and IVM species prevalence rates. This suggests the presence of infective larvae from the environment or recently emerged adults that would be detected by NGS methods and not by the McMaster FEC. The contrasting FECR and SFPR results observed in this study may be a reflection of quick reinfection by a contaminated pasture or the emergence of the encysted larvae from the mucosal tissues. The physiology underlying excystment rates and triggers are still undiscovered because of the difficulty of observing and noninvasively detecting encysted larvae [71,72].

The FEC method has been criticized for underestimating parasite burdens due to its inability to detect the larval population [71], whereas NGS can detect the genetic material of all lifecycle stages. However, a limitation of the molecular tools is that they cannot estimate parasite abundances because an individual can contribute multiple copies of the genomic material based on the lifecycle stage [52].

The preservation of the refugia population has been praised as a positive deworming management practice because the refugia have undergone less selective pressure for anthelmintic resistance and thus preserves the anthelmintic-sensitive genetics [1,30,70]. When the refugia population is lost, the genetic pool is dominated by highly resistant genetics.

### 4.2. Anthelmintic Resistance

Similar to our results, Lyons et al. [73] observed multidrug class resistance in *CT. goldi, CT. minutus,* and *CS. catinatum* in a herd of benzimidazole-resistant Shetland ponies following pyrantel and oxibendazole anthelmintics. The same study [73] also observed resistance *in CY. nassatus, CO. coronatus, CT. longibursatus,* and *CT. calicatus* that was not observed in the present study, although this study found that *CT. longibursatus* may be showing a sign of the beginning signs of resistance to PYR.

It is possible that correlations between species and treatments were only found in MOX because of this drug’s capacity to target encysted larvae reducing a higher proportion of the parasite burden when compared to IVM and PYR. Three of the five species that persisted following MOX treatment (*CY. leptostomus, CY. insigne,* and *CS. catinatum)* appear to be part of the ‘core’ group and most likely have higher anthelmintic resistance levels. Over time these species may have decreased due to prolonged exposure to MOX treatment and its increased lipophilicity when compared to PYR and IVM [70,74]. Interestingly, *CO. labratum* was found to be positively correlated with MOX treatment (r = 0.36). This could either indicate less efficacy of MOX to this species, differential host response, or a seasonal effect.

### 4.3. Phylogeny

It has been hypothesized that closely related species may be the next to acquire multidrug resistance [27,28]. Based on the phylogenetic relationships of the cyathostomin gene sequences, the results of this study suggest emerging resistance in *CY. auriculatus* and *CT. longibursatus*.

The inheritance of the anthelmintic resistance traits is not well understood in cyathostomins due to the lack of complete resistance and inadequate models. The genetics of resistance traits in rumen gut nematodes have been reported to be expressed as incomplete dominant, complete dominant, incomplete recessive, sex-linked recessive, and autosomal recessive and will vary between parasites and drugs [75]. The *H. contortus* (Trichostrongylidae) rumen parasite with complete anthelmintic resistance is a close relative to equine cyathostomins (Strongylidae) as members of the Strongylida order [76,77]. In *H. contortus*, mutations in multiple isotype-I and -II genes and GluCl channels may be responsible for IVM and bendazole resistance [13]. In equine cyathostomins, mutations of the isotype-I and -II genes have been found in bendazole and IVM-resistant *CY. nassatus* and *CS. catinatum*. *CS. catinatum, CS. tetracanthum, CY. nassatus, and CS. goldi* possess a GluCl α4 subunit but demonstrate a 12% inter-specific variation within the species with an additional 4% intra-specific variation within CY. nassatus [78,79]. This demonstrates that although there is low rDNA diversity between Strongylida taxa [42,80], there is large mDNA diversity at the species level [81]. The use of molecular methods enables noninvasive monitoring and tracking of resistance traits such as these.

## 5. Conclusions

Anthelmintic resistance has been a persistent problem for controlling cyathostomins in horses. It is imperative to slow the rate of resistance before complete resistance occurs. This study uses NGS profiling to demonstrate that there are species-specific differences between cyathostomins in response to anthelmintic treatment. Seven species were identified to demonstrate multidrug resistance and nine species to be acutely sensitive to MOX. MOX remains the most effective and PYR the least effective according to FECR and ERP measures but the most resistance is observed with IVM. Early detection of model organisms that demonstrate complete anthelmintic resistance will enable the discovery of genetic and ecophysiological differences between anthelmintic-sensitive and -resistant species to develop more targeted deworming strategies to control the resistant populations.

## Figures and Tables

**Table 1:** Description of horse subjects.

Horse ID	Sex ^1^	Age (Years)	Breed	Weight (kg)	Farm ID ^2^
1	G	10	Arabian	456	1
2	M	10	Arabian	449	1
3	G	8	Quarter Horse	600	1
4	M	10	Quarter Horse	534	1
5	G	7	Thoroughbred	490	1
6	G	16	Standardbred	470	1
7 *	G	19	Saddlebred	493	2 ^†^
8	M	18	Standardbred	498	2
9 *	G	33	Morgan	392	2

^1^ Sex is defined as gelding (G) or mare (M). ^2^ Farm 1 is located in Newark, DE, Farm 2 is located in Elkton, MD. * Horse 7 was not included in the sequencing data for MOX and Horse 9 for PYR due to unsuccessful amplification for >50% of the time points. ^†^ Farm 2 was eliminated from the IVM trial due to previous deworming within 180 d prior to trial enrollment.

**Table 2:** Species accession numbers of aligned sequences of cyathostomins for taxonomy assignments. The naming conventions of Lichtenfels et al. [34] were used in this paper.

Taxa	Accession Number
*Cylicocyclus (CY) ashworthi*	Y08586
*Cylicocyclus (CY) leptostomus*	KP693432
*Cylicocyclus (CY) nassatus*	Y08585
*Cylicocyclus (CY) radiatus*	JQ906423
*Cyathostomum (CS) tetracanthum*	KF850629
*Cylicocyclus (CY) insigne*	Y08588
*Cylicocyclus (CY) auriculatus*	JQ906414
*Coronocyclus (CO) labiatus*	JN786947
*Coronocyclus (CO) labratum*	AJ004838
*Cylicostephanus (CT) calicatus*	KM085356
*Cylicocylus (CY) elongatus*	JQ906417
*Cyathostomum (CS) pateratum*	KF850627
*Cyathostomum (CS) catinatum*	KF850626
*Cylicostephanus (CT) goldi*	KM085357
*Cylicostephanus (CT) longibursatus*	KM085358
*Cylicodontophorus (CD) bicoronatus*	KP693441
*Cylicostephanus (CT) minutus*	KM085361
*Coronocyclus (CO) coronatus*	JN786951
*Poteriostomum (POT) imparidentatum*	KP693433

**Table 3:** Reappearance of cyathostomins over 98 days post deworming with three different anthelmintics.

	FECR ^1^/ERP ^2^			Total Number of Species ^3^			
Day	IVM (%)	MOX (%)	PYR (%)	CON	IVM	MOX	PYR
0	--	--	--	13.16 ± 3.7	10.5 ± 5.4	10.9 ± 4.6	7.88 ± 4.6
14	100.00	100.00	98.37	13.16 ± 3.7	6.50 ± 7.2	7.13 ± 3.9	7.88 ± 4.1
28	96.15	100.00	72.29 *	9.00 ± 5.0	10.7 ± 5.4	5.13 ± 3.3	5.50 ± 4.8
42	81.73 *	98.58	49.46	9.00 ± 5.0	12.2 ± 2.6	5.63 ± 3.4	9.13 ± 4.9
56	53.85	96.45	7.63	14.33 ± 2.3	10.2 ± 5.7	4.25 ± 5.2	10.8 ± 4.1
70	67.31	93.85	−91.29	14.33 ± 2.3	12.0 ± 3.3	6.00 ± 3.7	11.8 ± 3.3
84	34.62	83.91 *	−152.69	10.3 ± 3.7	10.7 ± 5.1	4.75 ± 3.1	11.5 ± 4.4
98	0.00	60.25	−167.36	10.3 ± 5.7	9.33 ± 4.6	6.88 ± 6.2	9.75 ± 3.9

^1^ Fecal egg count reduction and ^2^ egg reappearance period. ^3^ Average number of the total species present at each timepoint ± the standard deviation. * indicates a shortened ERP using an FECR cut-off of ≤90% for IVM and MOX and ≤80% for PYR as outlined by Nielsen et al. [26]. CON–control (untreated), IVM–Ivermectin, MOX–Moxidectin, PYR–Pyrantel. The total number of species present per timepoint allows tracking of the total number of present cyathostomins to see if horses are reinfected by many species or specific species that increase numerically to make up the majority of the parasite burden.

**Table 4:** ANOVA and Spearman correlations based on frequency of presence.

					*p*-Value	Spearman Correlation (r)
Worm Species	CON	IVM	MOX	PYR	Treatment	MOX
*CO. coronatus*	1.00 ^a^	0.77 ^ac^	0.20 ^b^	0.64 ^c^	3.36 × 10^−6^ ***	−0.44
*CO. labiatus*	0.17 ^a^	0.08 ^a^	0.06 ^a^	0.04 ^a^	0.403	
*CO. labratum*	0.19 ^a^	0.04 ^a^	0.24 ^a^	2.7 × 10^−17 a^	0.038 *	0.36
*CS. catinatum*	1.00 ^a^	0.83 ^a^	0.56 ^a^	0.83 ^a^	0.066	
*CS. tetracanthum*	0.02 ^a^	0.0 ^a^	0.06 ^a^	0.00 ^a^	0.281	
*CY. ashworthi*	1.00 ^a^	0.83 ^a^	0.47 ^b^	0.75 ^a^	0.0004 ***	−0.38
*CY. auriculatus*	0.42 ^a^	0.31 ^a^	0.21 ^a,b^	0.24 ^a^	0.045 *	
*CY. insigne*	0.57 ^a^	0.52 ^a,c^	0.21 ^b^	0.46 ^b,c^	9.2 × 10^−^^5^ ***	−0.31
*CY. leptostomus*	0.76 ^a^	0.67 ^a^	0.27 ^b^	0.44 ^b^	1.13 × 10^−5^ ***	−0.32
*CY. nassatus*	0.85 ^a^	0.83 ^a^	0.47 ^b^	0.79 ^a^	3.04 × 10^−5^ ***	−0.34
*CY. radiatus*	0.97 ^a^	0.71 ^a^	0.33 ^b^	0.69 ^a^	0.0004 ***	−0.41
*CS. pateratum*	0.91 ^a^	0.83 ^a^	0.67 ^a^	0.81 ^a^	0.00159 ***	
*CY. elongatus*	0.56 ^a^	0.31 ^a^	0.22 ^a^	0.38 ^a^	0.106	
*CD. bicoronatus*	0.48 ^a^	0.42 ^a^	0.16 ^b^	0.36 ^a^	0.108	
*CT. calicatus*	0.97 ^a^	0.77 ^a,c^	0.36 ^b^	0.61 ^b,c^	0.0005 ***	−0.31
*CT. goldi*	0.92 ^a^	0.79 ^a^	0.74 ^a^	0.78 ^a^	0.189	
*CT. longibursatus*	1.00 ^a^	0.83 ^a,b^	0.71 ^b^	0.89 ^a,b^	0.025 *	
*CT. minutus*	0.57 ^a^	0.54 ^a^	0.13 ^b^	0.46 ^a^	0.074	−0.36

CON–control; MOX–Moxidectin; IVM–Ivermectin; PYR–Pyrantel. Superscripts with different letters within row demonstrate *p* < 0.05 with Tukey’s all-pair comparison testing. Spearman correlations for IVM and PYR are not shown because no significant correlations were found. ANOVA significance was determined at (*p* ≤ 0.05) and a tendency toward significance at (0.05 ≤ *p* ≥ 0.10). Spearman’s coefficient, r, significance was determined at (0.3 ≤ r ≥ −0.3). The Spearman correlation, r, is considered to be fairly significant at (≥+/−0.3–<+/−0.5), moderate with (≥+/−0.5–<+/−0.7), strong with (≥+/−0.7–<+/−0.9), and substantial with (≥ +/−0.9–+/−1.0).

## Data Availability

Sequence data has been submitted to the NCBI Sequence Read Archive within PRJNA716069.

## Supplementary Materials

The following are available online at https://www.mdpi.com/article/10.3390/ani11051345/s1, Table S1: Description of CON horse subjects, Table S2: Comparison of morphological and molecular ID of cyathostomin species, Table S3: Frequency of presence of cyathostomins following treatment, Table S4: Total species variation per horse at Day 0, Table S5: Species prevalence in CON samples.
