# Supplementary material for: The Use of Molecular Profiling to Track Equine Reinfection Rates of Cyathostomin Species Following Anthelmintic Administration
Source: Animals (Basel). 2021 May 9;11(5):1345. doi: 10.3390/ani11051345 (PMC8150961; doi:10.3390/ani11051345)
Supplement: Supplementary file 1 [file animals-11-01345-s001.zip › animals-1174031-supplementary.pdf]

Supplementary Tables:

Table S1: Description of CON horse subjects

Day[1]	Fecal samples (n)[2]	FEC (epg)[3]	Farms (#)[4]
0	12	700	3
14	12	700	3
28	14	277	2
42	14	299	2
56	9	--	3
70	9	--	3
84	10	433	3
98	10	506	3
**Totals**	**90**	**433* (avg)**	**7**

Table S1: [1] – Day represents the timepoint of horses enrolled in the study that the CON sample corresponds with. [2] – Fecal samples lists the number of samples obtained for each study timepoint. [3] – Fecal egg counts performed with the modified McMaster technique and represented as eggs per gram (epg). CON samples that correspond with Day 70 and 84 are not shown due to missing data. [4] – Farms lists the total number of farms that samples were obtained from.

Table S2: Comparison of morphological and molecular ID of cyathostomin species

Sample Name.	Morphological ID	Molecular ID	Pairwise ID[2] (%)
U1	*CS. catinatum*	*CS. catinatum*	98.6
U2	*CS. catinatum*	*CS. catinatum*	99.2
U3	*CT. goldi*	*CT. goldi*	97.1
U4	*CS. catinatum*	*CS. catinatum*	61.3
U5	*CO. labratum*	*CO. labratum*	64.4
U6	*CS. catinatum*	*CS. catinatum*	74.6
U7	[1]	*CT. minutus*	64.6
U9*	*CT. goldi*	*CO. labiatus*	52.0
U10	*CS. catinatum*	*CS. catinatum*	62.4
U11	*CT. longibursatus*	*CT. longibursatus*	88.9
U12	[1]	*CT. goldi*	94.1
U13	*CT. goldi*	*CT. goldi*	96.1
U14	*CS. catinatum*	*CS. catinatum*	99.0
U15	*CS. catinatum*	*CS. catinatum*	85.8
U16	*CS. catinatum*	*CS. catinatum*	98.3
U17	*CS. catinatum*	*CS. catinatum*	99.2
U18	*CS. catinatum*	*CS. catinatum*	98.1
U19	*CS. catinatum*	*CS. catinatum*	99.0
U20[†]	*CO. labiatus*	*CS. pateratum*	85.6

Table S2: Samples were independently identified using correlative molecular and morphological methods. In two cases, molecular ID incorrectly identified cyathostomin species compared to morphological ID. *U9 had a low percentage of identical sites which could have been due to non-specific base pairing in key sections of the gene that caused this sequence to be so varied. The low pairwise identity (52%) of U9 to the reference file could indicate poor quality of DNA or sequencing error. [†] Morphologic ID incorrectly identified cyathostomin species compared to molecular ID. U20 was "an uncommon L4/L5 stage in which the buccal capsule of the fourth stage larva is still visible within the sheath being shed by the emerging young adult" (personal communication, CRR). Thus, illustrating the difficulty of conclusively identifying cyathostomins by phenotypic traits. Furthermore, a Maximum Likelihood tree (Boostrap analysis of 500 replicates) showed the close clustering (bootstrap value = 0.668) of U20 with *CS. pateratum* (Data not shown). [1] No morphological identification could be made on samples. 2 Pairwise ID% indicates the level of similarity between Sample and NCBI reference sequences listed in Table 2.

Table S3: Frequency of presence of cyathostomins following treatment

	Day								P-value		
	**0**	**14**	**28**	**42**	**56**	**70**	**84**	**98**	**Trt[1]**	**Day**	**TxD[2]**
CO. coronatus									<0.001	0.07	0.11
CON[3]	0.82	0.85	0.57	0.57	1.00	1.00	0.70	0.70			
MOX[4]	0.63	0.00	0.00	0.13	0.13	0.13	0.13	0.50			
IVM[5]	0.83	0.50	0.67	1.00	0.56	1.00	0.83	0.67			
PYR[6]	0.38	0.63	0.38	0.75	0.75	0.88	0.75	0.63			
**P-value**	**0.15**	**0.001**	**0.03**	**0.008**	**<0.001**	**<0.001**	**0.01**	**0.90**			
CO. labiatus									0.08	0.14	0.17
CON	0.25	0.25	0.07	0.07	0.33	0.33	0.00	0.00			
MOX	0.25	0.00	0.00	0.00	0.13	0.11	0.00	0.00			
IVM	0.00	0.00	0.00	0.33	0.00	0.17	0.17	0.00			
PYR	0.13	0.00	0.00	0.00	0.00	0.0	0.25	0.00			
**P-value**	**0.57**	**0.11**	**0.68**	**0.10**	**0.15**	**0.34**	**0.23**	**--**			
CO. labratum									<0.001	0.004	<0.001
CON	0.45	0.38	0.00	0.00	0.44	0.44	0.00	0.00			
MOX	0.44	0.63	0.63	0.00	0.00	0.25	0.00	0.00			
IVM	0.00	0.00	0.17	0.17	0.00	0.00	0.00	0.00			
PYR	0.00	0.00	0.00	0.00	0.00	0.00	0.00	0.00			
**P-value**	**0.03**	**0.008**	**<0.001**	**0.34**	**0.07**	**0.07**	**0.10**	**--**			
CS. catinatum									0.03	0.59	0.25
CON	1.00	1.00	0.79	0.79	1.00	1.00	0.90	0.90			
MOX	1.00	0.88	0.75	0.00	0.00	0.75	0.50	0.63			
IVM	0.83	0.50	0.83	1.00	0.83	1.00	0.83	0.83			
PYR	0.63	0.63	0.63	0.88	1.00	0.88	1.00	1.00			
**P-value**	**0.18**	**0.06**	**0.83**	**0.62**	**0.07**	**0.31**	**0.06**	**0.50**			
CS. tetracanthum									0.05	0.09	0.004
CON	0.08	0.08	0.00	0.00	0.00	0.00	0.00	0.00			
MOX	0.00	0.00	0.00	0.00	0.10	0.38	0.00	0.00			
IVM	0.00	0.00	0.00	0.00	0.00	0.00	0.00	0.00			
PYR	0.00	0.00	0.00	0.00	0.00	0.00	0.00	0.00			
**P-value**	**0.63**	**0.63**	**0.0**	**0.0**	**0.43**	**0.02**	**0.00**	**0.00**			
CY. ashworthi									<0.001	0.22	0.16
CON	1.00	1.00	0.86	0.86	1.00	1.00	0.80	0.80			
MOX	0.88	0.63	0.25	0.50	0.25	0.25	0.38	0.63			
IVM	0.83	0.50	0.83	1.00	0.83	1.00	0.83	0.83			
PYR	0.88	0.63	0.50	0.75	0.88	0.88	0.88	0.75			
**P-value**	**0.63**	**0.07**	**0.02**	**0.13**	**<0.001**	**<0.001**	**0.10**	**0.82**			
CY. auriculatus									0.034	0.05	0.68
CON	0.58	0.58	0.21	0.21	0.67	0.67	0.20	0.20			
MOX	0.25	0.25	0.13	0.13	0.13	0.50	0.00	0.25			
IVM	0.33	0.33	0.33	0.17	0.33	0.33	0.17	0.50			
PYR	0.13	0.25	0.25	0.38	0.25	0.38	0.25	0.00			
**P-value**	**0.18**	**0.37**	**0.84**	**0.68**	**0.11**	**0.58**	**0.57**	**0.18**			
CY. insigne									<0.001	0.19	0.34
CON	0.67	0.67	0.36	0.36	0.78	0.78	0.50	0.50			

MOX	0.38	0.38	0.13	0.25	0.13	0.25	0.00	0.13			
IVM	0.50	0.33	0.67	0.50	0.50	0.67	0.50	0.50			
PYR	0.25	0.13	0.15	0.50	0.50	0.88	0.75	0.38			
**P-value**	**0.32**	**0.11**	**0.20**	**0.72**	**0.06**	**0.04**	**0.02**	**0.39**			
CY. leptostomus									<0.001	0.002	0.17
CON	0.92	0.92	0.64	0.64	1.00	1.00	0.50	0.50			
MOX	0.75	0.25	0.13	0.13	0.00	0.38	0.38	0.25			
IVM	0.83	0.33	0.67	0.83	0.67	0.83	0.67	0.50			
PYR	0.50	0.38	0.13	0.50	0.63	0.88	0.50	0.38			
**P-value**	**0.20**	**0.006**	**0.01**	**0.04**	**<0.001**	**0.01**	**0.79**	**0.73**			
CY. nassatus									<0.001	0.60	0.88
CON	0.75	0.75	0.79	0.79	1.00	1.00	0.90	0.90			
MOX	0.50	0.63	0.25	0.50	0.50	0.25	0.63	0.50			
IVM	0.83	0.50	0.83	1.0	0.83	1.00	0.83	0.83			
PYR	0.75	0.75	0.63	0.75	0.88	0.88	0.75	0.88			
**P-value**	**0.55**	**0.72**	**0.05**	**0.20**	**0.06**	**<0.001**	**0.58**	**0.18**			
CY. radiatus									<0.001	0.62	0.61
CON	0.83	0.83	0.86	0.86	0.89	0.89	0.80	0.80			
MOX	0.63	0.50	0.13	0.25	0.13	0.13	0.38	0.50			
IVM	0.83	0.50	0.83	0.83	0.67	0.83	0.83	0.67			
PYR	0.88	0.63	0.38	0.63	0.75	0.88	0.75	0.75			
**P-value**	**0.63**	**0.40**	**0.001**	**0.02**	**0.005**	**<0.001**	**0.19**	**0.60**			
CS. Pateratum									0.002	0.55	0.03
CON	1.00	1.00	0.79	0.79	1.00	1.00	0.90	0.90			
MOX	1.00	0.75	0.75	0.88	0.38	0.38	0.63	0.63			
IVM	0.83	0.50	0.83	1.00	0.83	1.00	0.83	0.83			
PYR	0.75	0.75	0.50	0.88	0.88	1.00	0.88	0.88			
**P-value**	**0.18**	**0.09**	**0.48**	**0.68**	**0.01**	**<0.001**	**0.50**	**0.50**			
CY. elongatus									0.0006	0.007	0.06
CON	0.67	0.67	0.29	0.29	1.00	1.00	0.30	0.30			
MOX	0.38	0.13	0.0	0.13	0.13	0.38	0.25	0.38			
IVM	0.33	0.33	0.50	0.50	0.50	0.33	0.50	0.17			
PYR	0.25	0.13	0.25	0.38	0.50	0.63	0.63	0.25			
**P-value**	**0.27**	**0.03**	**0.20**	**0.50**	**0.001**	**0.02**	**0.41**	**0.87**			
CD. bicoronatus									<0.001	0.02	0.65
CON	0.58	0.58	0.29	0.29	0.56	0.56	0.50	0.50			
MOX	0.50	0.00	0.00	0.00	0.13	0.25	0.00	0.38			
IVM	0.50	0.17	0.17	0.33	0.50	0.50	0.50	0.17			
PYR	0.13	0.25	0.00	0.25	0.50	0.50	0.50	0.63			
**P-value**	**0.24**	**0.03**	**0.17**	**0.41**	**0.30**	**0.64**	**0.10**	**0.39**			
CT. calicatus									<0.001	0.64	0.11
CON	0.83	0.83	0.57	0.57	0.89	0.89	0.80	0.80			
MOX	0.75	0.50	0.50	0.13	0.13	0.25	0.13	0.50			
IVM	0.83	0.50	0.83	1.00	0.83	0.67	0.83	0.67			
PYR	0.38	0.63	0.38	0.50	0.75	0.75	0.75	0.75			
**P-value**	**0.13**	**0.40**	**0.41**	**0.009**	**0.002**	**0.04**	**0.005**	**0.60**			
CT. goldi									0.04	0.43	0.06
CON	1.00	1.00	0.71	0.71	1.00	1.00	1.00	1.00			

MOX	1.00	0.88	0.75	0.88	0.50	0.75	0.63	0.63			
IVM	0.83	0.50	0.83	1.00	0.83	0.83	0.67	0.83			
PYR	0.63	0.75	0.50	0.88	0.88	0.88	0.88	1.00			
**P-value**	**0.04**	**0.06**	**0.58**	**0.45**	**0.06**	**0.52**	**0.17**	**0.06**			
CT. longibursatus											
CON	1.00	1.00	0.86	0.86	1.00	1.00	1.00	1.00	<0.001	0.47	0.14
MOX	1.00	0.88	0.75	0.63	0.50	0.63	0.75	0.63			
IVM	0.83	0.50	0.83	1.00	0.83	1.00	0.83	0.83			
PYR	0.88	0.88	0.63	0.88	1.00	1.00	1.00	1.00			
**P-value**	**0.40**	**0.04**	**0.66**	**0.30**	**0.01**	**0.02**	**0.23**	**0.06**			
CT. minutus									<0.001	0.08	0.10
CON	0.67	0.67	0.36	0.36	0.78	0.78	0.50	0.50			
MOX	0.50	0.00	0.00	0.00	0.13	0.00	0.00	0.38			
IVM	0.50	0.50	0.83	0.50	0.50	0.83	0.83	0.50			
PYR	0.25	0.25	0.13	0.38	0.63	0.63	0.75	0.63			
**P-value**	**0.37**	**0.01**	**0.003**	**0.19**	**0.05**	**0.001**	**0.002**	**0.82**			

Table S3: [1] – Treatment, [2] – Interaction of treatment and day, [3] – Control, [4] – Moxidectin, [5] – Ivermectin, [6] – Pyrantel

Table S4: Total species variation per horse at Day 0

Horse ID	N	Total Number of Species	SD
1	4	8	5.66
2	4	13.75	3.86
3	4	11.25	1.26
4	4	11.5	2.38
5	4	6.5	4.43
6	4	8	3.83
7	1	4	NA
8	2	16.5	0.71
9	1	5	NA

Table S4: N – number of observations. SD – Standard deviation. Descriptive data of horses can be found in Table 1.

Table S5: Species prevalence in CON samples

		Chapman et al., [5]	
**Species**	**CON Prevalence (rank)**	**2002 rank**	**1986 rank**
CT. longibursatus	94.12 (1)	1	1
CS. catinatum	89.92 (2)	2	3
CS. pateratum	89.92 (3)	13	11
CY. ashworthi	89.08 (4)	11	7

CT. goldi	89.08 (5)	4	6
CY. radiatus	82.35 (6)	17*	16
CY. nassatus	80.67 (7)	5	5
CT. calicatus	73.95 (8)	6	8
CY. leptostomus	73.11 (9)	9	4
CO. coronatus	71.43 (10)	7	9
CT. minutus	53.78 (11)	3	2
CY. insigne	52.94 (12)	8	10
CY. elongatus	50.42 (13)	16*	17
CD. bicoronatus	46.22 (14)	20*	15
CY. auriculatus	37.82 (15)	NA	NA
CO. labratum	19.33 (16)	10	13
CO. labiatus	15.13 (17)	12	12
CS. tetracanthum	1.68 (18)	NA	NA

Table S5: Comparison of species prevalence found in this study compared to findings of Chapman et al., [5]. Chapman et al., [5] reports cyathostomin species prevalence from 2002 and 1986 of ponies with minimal exposure to anthelmintics in Louisiana. Chapman et al., [5] morphologically identified 100 worms following necropsy. While the methods differ between the results of this study and that of Chapman et al., [5], the molecular tools used in this study is able to identify a similar core group of cyathostomins as morphological identification methods finds. * 2002 and 1986 surveys included other cyathostomin species not surveyed in this study so ranks #15, 18, 19 are skipped. NA – species were not included in the 2002 and 1986 surveys.
